# Duration of Analgesia Induced by Acupuncture-Like TENS on Experimental Heat Pain

**DOI:** 10.1155/2013/792383

**Published:** 2013-04-07

**Authors:** Yannick Tousignant-Laflamme, Marilyne Brochu, Cynthia Dupuis-Michaud, Catherine Pagé, Draga Popovic, Marie-Eve Simard

**Affiliations:** ^1^École de Réadaptation, Faculté de Médecine et des Sciences de la Santé, Université de Sherbrooke, Pavillon Gérald-Lasalle, Z7-2011, 3001, 12e Avenue Nord, Sherbrooke, QC, Canada J1H 5N4; ^2^Centre de Recherche Clinique Etienne-LeBel du CHUS, Sherbrooke, QC, Canada J1H 5N4

## Abstract

*Background*. Acupuncture-like TENS (AL-TENS) is a treatment modality that can be used to temporarily reduce pain. However, there is no clear data in the literature regarding the specific duration of analgesia induced by AL-TENS. *Objectives*. To describe and quantify the duration and magnitude of AL-TENS analgesia on experimental heat pain in healthy subjects and verify if the duration or magnitude of analgesia induced by the AL-TENS was influenced by the duration of the application of the AL-TENS (15 versus 30 minutes). *Methods*. A repeated-measures, intrasubject randomized experimental design was used, where each participant was his/her own control. 22 healthy volunteers underwent heat pain stimulations with a contact thermode before (pretest) and after (posttest) AL-TENS application (15 and 30 minutes). Outcome measures included subjective pain during AL-TENS, duration, and magnitude of AL-TENS-induced analgesia. *Results*. Survival analysis showed that the median duration of AL-TENS analgesia was 10 minutes following the application of either 15 or 30 minutes of AL-TENS. The magnitude of analgesia following either application was comparable at all points in time (*P* values > 0.05) and ranged between −20% and −36% pain reduction. *Conclusion*. Only half of the participants still had heat-pain analgesia induced by the AL-TENS at 15 minutes postapplication.

## 1. Introduction

Pain is one of the principal motives to consult a health care professional [1]. Among the analgesic modalities used by rehabilitation professionals, transcutaneous electrical nerve stimulation (TENS) is commonly used, as it is noninvasive, safe, and affordable; it has no undesirable side effects and can be easily administrated in clinical settings as well as by the patients themselves [2].

During or following TENS application, categorized as either *Conventional TENS *or *Acupuncture-like TENS *(AL-TENS), analgesia is induced by different pain control mechanisms depending on the parameters used. Parameters for conventional TENS include high-frequency (50–100 Hz), low-intensity (nonpainful paraesthesias) stimulations, and a small pulse width (50–200 *μ*s). On the other hand, AL-TENS parameters include low-frequency (2–10 Hz), high-intensity (above pain threshold) stimulations, and a longer pulse width (100–400 *μ*s). Conventional TENS analgesia is explained by the gate control theory [3, 4], whereas the AL-TENS pain modulation mechanism is explained by descending endogenous opiate system [5].

The later pain modulation mechanism modulates nociceptive transmission by inhibiting ascending nociceptive pathways in the dorsal horn of the spine at multiple segmental levels as a result of descending efferents [6–8]. Although other pain control mechanisms might be involved in AL-TENS, such as GABA and serotonin (demonstrated in animal models) [9], muscle contraction [10], the main analgesic effect arises from this classic opioid-dependent descending inhibitory pathway.

Although the pain modulation mechanisms involved in AL-TENS are well known, there is limited evidence in the literature regarding the duration of analgesia induced by AL-TENS. The current data is arising from clinical studies [8, 11–13], studies involving experimental pain stimuli in healthy subjects [14–18], and animal studies [19–21]. The results range from 30 minutes to “at least an hour” postapplication and it is generally accepted that AL-TENS analgesia is of longer duration than the analgesia induced using the conventional TENS [11]. However, the results regarding the duration of AL-TENS pain modulation in the above studies have to be interpreted with caution since the duration of analgesia was not the main outcome tested in any of these studies.

A better understanding of AL-TENS analgesia, specifically duration, would be beneficial since the duration of the analgesia induced by AL-TENS can influence the therapeutic options chosen by the rehabilitation professionals (i.e., AL-TENS versus conventional TENS). With this in mind, the main objective of this study was to describe the duration of analgesia and magnitude of analgesia induced by the application of AL-TENS on healthy subjects with an experimentally induced heat pain stimulus. The secondary objective was to verify if the duration or magnitude of analgesia induced by the AL-TENS was influenced by the duration of the application of the AL-TENS (15 and 30 minutes).

## 2. Methodology

### 2.1. Design

A repeated-measures, intrasubject randomized experimental design was used, where each participant was his/her own control. Participants were randomly assigned to either begin with the 15 (*T*_15_) or 30 (*T*_30_) minutes application of AL-TENS. This counterbalancing of participants minimized a potential sequence effect. Since this was an intrasubject design, concealment of allocation was not possible—all subjects knew that they would receive the 15 or 30 minutes treatment during the second session.

### 2.2. Participants

The study was conducted in accordance with the Helsinki Declaration, and after approval from the ethics review board of the *Centre de recherche clinique Étienne LeBel* (CHUS); data was collected from 22 healthy volunteers, 8 men and 14 women. The mean age of our participants was 25.41 ± 9.33 years. Inclusion criteria were healthy and pain-free volunteers aged 18 years or above, being able to understand and follow instructions. Volunteers were excluded if they were presenting any painful pathology, loss of sensibility to heat, and pain on treatment areas or had any contraindication to AL-TENS (i.e., wearing a pacemaker or cardioverter defibrillator, wearing a metal implant under the treatment area by the AL-TENS, suffering of epilepsy, and presenting a haemorrhagic risk, a malignancy, or damaged skin under the treatment area) [22]. All participants were advised to avoid any kind of analgesic medication for a period of 12 hours before the experimentation and avoid caffeinated beverages 4-5 hours before experimentation since caffeine can block the analgesic effect of TENS [23, 24]. All participants were recruited on a voluntary basis and were naïve to AL-TENS. All subjects signed an informed consent form and received a free parking voucher for each visit. Each experimental procedure lasted about 90 minutes.

### 2.3. Data Collection Process

The study took place at the School of Rehabilitation of the *Université de Sherbrooke* between January 2011 and November 2011. Five physiotherapy students involved in this research project carried out the experiments. All five students were trained by the principal investigator (YTL) in order to standardize the data collection process. All participants received the experimental procedures in the same sequential order; familiarization, determination of test-stimulus intensity, test stimulus, AL-TENS application, and assessment of AL-TENS analgesia. All subjects received AL-TENS application in two separate sessions (on different days) of either 15 or 30 minutes duration by random assignment. We arbitrarily chose 15 and 30 minutes treatment times since they are the typical treatment durations [22]. Both experiments were separated by at least 6 days to limit any possible carryover effect from session 1 to session 2. 

### 2.4. Heat Pain Procedures

A sustained (tonic) experimental heat pain test was chosen since it is safe, easily reproducible and may be more comparable to clinical pain than to brief (phasic) pain tests [25].

#### 2.4.1. Familiarization Period

The noxious stimulus was induced by a 9 cm^2^ thermode (TSA II, NeuroSensory Analyzer, Medoc Instruments, North Carolina, USA). They were then familiarized with the experimental (thermal) pain (test stimulus) induced by the thermode on the palm of the dominant hand. Subjects were advised that the thermode temperature would gradually increase to a maximum of 51°C and reassured that it could not induce any burn. This procedure was repeated twice and the subjects had to verbally report when they began to feel pain (pain threshold) and when the pain was intolerable (pain tolerance).

#### 2.4.2. Determination of the Test-Stimulus Intensity

To determine the test-stimulus intensity (temperature of heat pain), the thermode was applied on the volar aspect of the dominant forearm. Pain perception was assessed with a computerized visual analogue scale (COVAS) graduated from 0 (no pain) to 100 (maximum tolerable pain) linked to the thermode. This instrument has a good validity and reliability and was previously used in other experimental pain studies [26]. The subjects had to move the cursor when they reported initial pain sensation (COVAS score: 1/100 which corresponds to the pain threshold). As the temperature of the thermode increased, subjects moved the cursor towards the right. When the maximum temperature they could tolerate was reached, the cursor needed to be at the extreme right (COVAS score: 100/100 which corresponds to the pain tolerance). The experimenter then immediately turned off the thermode. Between the trials, the thermode was placed on another area of the forearm to avoid overstimulation and possible local sensitization. Subjects were given at least two trials of test stimulus to practice heat pain ratings with the COVAS and to ensure consistent responses between trials. Then, the temperature rated as 50/100 on the COVAS was used for the next part of the experimentation, the test stimulus (one-minute constant stimulation).

#### 2.4.3. Test Stimulus

For the test stimulus, the thermode was applied to the volar aspect of the dominant forearm one minute at the temperature corresponding to 50/100 pain perception on COVAS. Subjects were told that they had to move the cursor left or right as decreased function of the pain perceived. In this part of the test, the temperature always increased to reach the temperature corresponding to 50/100 pain perception and then remained constant for one minute. All subjects were blinded to the temperature used during the test stimulus and to the study's hypothesis. The pain perception within this period had to be between 40/100 and 60/100 on the COVAS. Below or above these values, the temperature of the thermode was adjusted ±0.5°C to fit in this reasonable interval. When the temperature of the thermode produced a pain perception of approximately 50/100, AL-TENS was then applied.

#### 2.4.4. Induction of AL-TENS Analgesia (Conditioning Stimulus)

The AL-TENS (Eclipse, EMPI) was applied on the posterolateral aspect of the nondominant shoulder of all patients with the following parameters: 2 Hz frequency and 180 msec impulse duration (1 channel, 2 × 2 inches nonadhesive, reusable, black carbon rubber electrodes). This specific location was chosen for two reasons: (1) for standardization across all participants and most importantly (2) for demonstrating that AL-TENS induces a diffuse analgesic effect rather than a gate-control (segmental) analgesic effect. The pain intensity and unpleasant perception were recorded at 2 minutes intervals during the AL-TENS application by a numerical verbal scale ranging from 0 (no pain) to 100 (maximum tolerable pain). Since AL-TENS application needs to be painful in order to induce analgesia, the intensity of AL-TENS was increased until the subjects reported a perceived pain of at least 30/100 with the verbal numeric pain scale since it is shown that this type of descending analgesia can only be activated by a noxious stimulus [27]. 

### 2.5. Assessment of AL-TENS Analgesia

#### 2.5.1. Duration

To measure the duration of AL-TENS analgesia, series of one minute test stimulus were performed with the contact thermode one minute after the AL-TENS application and repeated at five minutes intervals until the mean perceived pain intensity was equal or above the perceived heat pain during the first test stimulus (before AL-TENS application). When this occurred, we considered that there was no more analgesia induced by AL-TENS and the experiment was terminated. The thermode was placed on another area of the dominant forearm between each repetition to avoid overstimulation.

#### 2.5.2. Magnitude

to measure the magnitude of AL-TENS analgesia (the amount of pain modulation produced by AL-TENS), we calculated the difference between the mean pain intensity score of the heat pain stimulus before and after the AL-TENS. A negative score indicated a reduction in pain perception and therefore analgesia. We considered that analgesia was no longer present when individual mean pain ratings of the post-AL-TENS test stimulus were above minus 0/100 (or a positive score). 

### 2.6. Statistical Analysis

Since our data was not normally distributed, nonparametric tests were used. Descriptive statistics are presented as median and interquartile range (IQR) [25th and 75th percentile] in the text and as median and standard error in the figures. In order to measure the duration of analgesia induced by the AL-TENS, a Kaplan-Meier survival analysis was used, with median duration as well as 95% confidence intervals. The McNemar test (non parametric) was used to determine if the duration of application of the AL-TENS (15 or 30 minutes) influenced the duration and/or magnitude of analgesia induced by the AL-TENS. A *P* value of less than 0.05 was considered statistically significant. SPSS software (version 18.0) was used to analyze our data.

## 3. Results

### 3.1. Pain Perception during AL-TENS Application

During the 15 minutes application of AL-TENS, the mean pain intensity was 34.12 ± 17.7/100 and the mean pain unpleasantness 58.12 ± 20.9/100. For the 30 minutes application of AL-TENS, the mean pain intensity was 31.95 ± 16.0/100 and the mean pain unpleasantness 58.46±23.6/100. Pain ratings for both treatment times were not statistically different.

### 3.2. Duration of AL-TENS Analgesia

**Figure 1:**
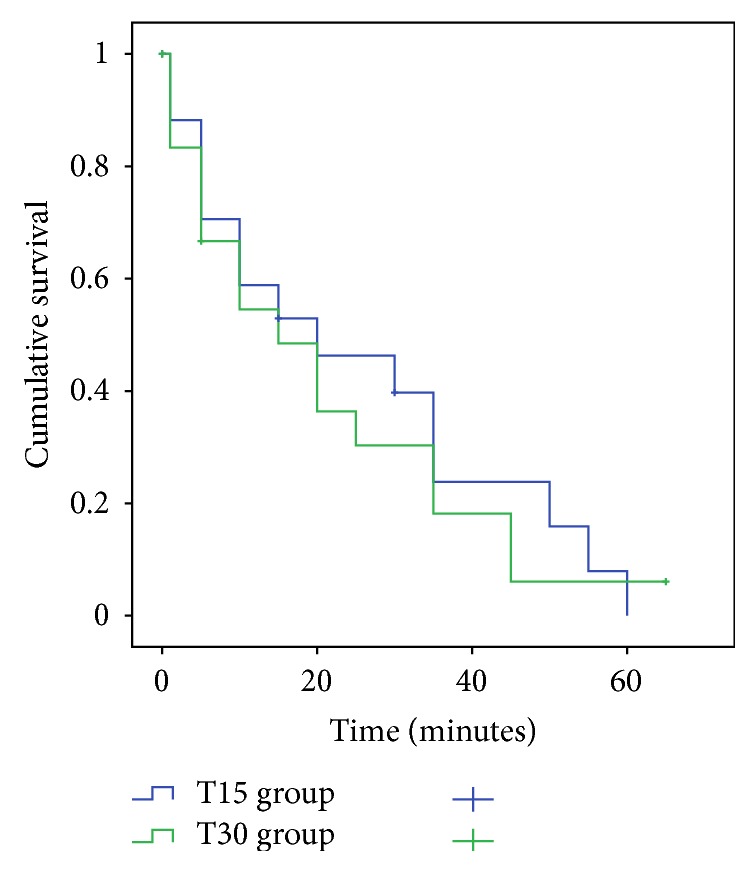
Survival analysis of analgesia induced by AL-TENS.

The median duration of the analgesia induced by AL-TENS was 10 minutes following both applications of either 15 or 30 minutes of AL-TENS (*T*_15_ = 10,00 [1,68–18,31]; *T*_30_ = 10,00 [0,00–23,58]). The analgesia induced by AL-TENS ranged from 0–60 minutes for the 15 minutes application and from 0–80 minutes for the 30 minutes application. Figure 1 illustrates the number of subjects that still had analgesia (analgesia versus no analgesia criteria) through time for both treatment times. The survival analysis performed showed no significant difference in the duration of analgesia between the 15 minutes and the 30 minutes application of AL-TENS as the two curves intersect throughout the graph (*P* = 0.991). Only half of the participants still had heat-pain analgesia induced by the AL-TENS at 15 minutes postapplication following both *T*_15_ and *T*_30_ groups (Figure 1). Finally, we observed that AL-TENS application did not induce analgesia in all participants. There were 3 participants in each group, the same subjects in the *T*_15_ and the *T*_30_ groups, who did not have a pain reduction during the first test stimulus after AL-TENS application.

### 3.3. Magnitude of AL-TENS Analgesia

**Figure 2:**
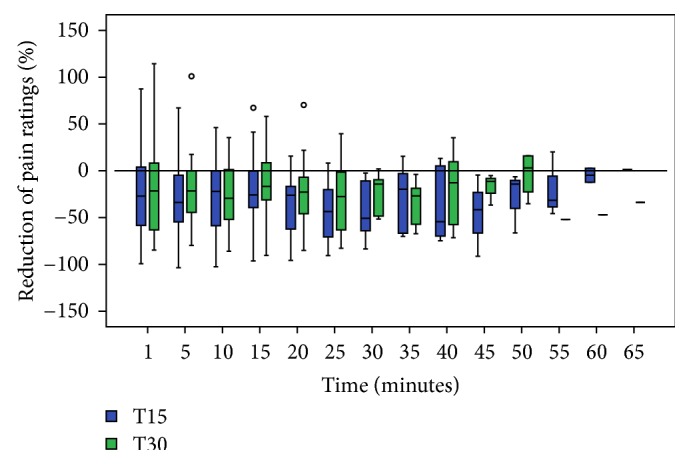
Comparison of the magnitude of AL-TENS analgesia (in reduction % of pain intensity ratings) following the application of AL-TENS (Box and Whisker chart).

The magnitude of AL-TENS analgesia was calculated for each 1-minute test stimulus following the AL-TENS application for the first 15 minutes post-TENS application since more than 50% of the subjects did not show analgesia after this point. We observed a statistically significant reduction in pain ratings after the application of AL-TENS (*P* = 0.016 and *P* = 0.03 for the *T*_15_ and *T*_30_ groups, resp.). The percentage of reduction in pain intensity for the *T*_15_ group ranged between −26% and −36% (Figure 2). Similarly, the percentage of reduction in pain for the *T*_30_ group during the same period ranged between −20% and −31%. No participant had a complete analgesia (100% in reduction pain ratings) with AL-TENS, as presented in Figure 2. We observed that either 15 or 30 minutes application of AL-TENS induced comparable magnitudes of analgesia at all points in time (all *P* values > 0.05) (see Figure 2).

## 4. Discussion

This study used a standardized experimental pain procedure and an intrasubject design to specifically determine the duration of the analgesia induced by AL-TENS. According to our findings, the median duration of AL-TENS analgesia on experimental heat pain was 10 minutes. Although the maximum duration was 80 minutes for the *T*_30_ and 60 minutes for the *T*_15_, the median durations for the two treatments time were comparable. Some experimental studies point out that at higher intensities AL-TENS produces inhibition of central nociceptive transmission for at least 1 hour [8, 13, 16, 19, 20]. The duration of analgesia we observed was much shorter, considering that it lasted less than 15 minutes for 50% of the sample as shown by the survival analysis. One previous study [28] explored the duration of noxious conditioning pain modulation (which relies on the same pain modulation mechanism) on healthy volunteers where the noxious conditioning pain was the cold pressor test (CPT). In this study, the mean duration of CPM analgesia was 35 minutes following the 7°C CPT and 10 minutes following the 12°C CPT. These results regarding the duration of analgesia are within the same range as we observed and further suggests that descending inhibition may represent an all-or-none phenomenon. This is not surprising given that comparable methodology and population were used to assess the duration and magnitude of the CPM. 

As part of the second objective, we wanted to determine if the duration of application of the AL-TENS (15 versus 30 minutes) influenced the duration of analgesia. Our survival analysis showed no significant difference between the analgesia duration following the 15 or 30 minutes application of AL-TENS. No previous study tried to determine if a longer application time of AL-TENS produced longer lasting analgesia. Since the duration of AL-TENS application did not seem to influence the analgesia duration, we can suppose that 15 minutes of AL-TENS application would be enough to produce analgesia which would allow the clinician (i.e., physical therapist) to maximize the efficiency of his intervention. As we observed (see Figure 1), it seems that the application of AL-TENS for 30 minutes tends to produce longer analgesia duration for some patients. However, these results were not statistically significant.

Along with the duration, we wanted to determine if the magnitude of analgesia induced by the AL-TENS was influenced by the duration of application of the AL-TENS (15 and 30 minutes). As expected with AL-TENS application, we observed a reduction in the perceived pain intensity with the application of either 15 or 30 minutes of AL-TENS [17]. However, there was no significant difference in the percentage of reduction of pain intensity between the 15 and 30 minutes application and this at each observation point after AL-TENS application. Furthermore, we found a large variation in the magnitude of reduction in pain perception between subjects. For example, in the *T*_15_ group the reduction between subjects varied between −67% and −10% at the 10 minutes observation. This demonstrates that the general response to AL-TENS analgesia varies among individuals. However, the large variability we observed in the magnitude of analgesia might explain the fact that the results were comparable at all points in time. No previous studies specifically investigated and compared the magnitude of AL-TENS analgesia.

### 4.1. Strength of the Study

This is the first study to explore the analgesia duration of AL-TENS with standardized protocol of experimental pain, which assure that all participants received comparable noxious stimuli during AL-TENS. Furthermore, we used an intrasubject design, which compares the results of the same participants following a 15 and a 30 minutes application of AL-TENS, where each participant was his own control. This reduces the interindividual variability.

### 4.2. Limitations of the Study

The main limitation of this study is linked to the fact that it was done on healthy subjects and used an experimental pain model that does not mimic clinical pain; it is wise not to extrapolate the present findings to clinical use and therefore the external validity is limited. Another limitation of this study is that most of the 22 participants were young (mean age 25.41 ± 9.33 years old) ascribable to a possible voluntary response bias. However, the participants were blinded to the hypothesis of the study. 

## 5. Conclusion

The application of AL-TENS on the shoulder above the pain threshold reduced perceived pain intensity of experimental heat pain at the dominant forearm in healthy participants for approximately 10 minutes. The analgesia induced by AL-TENS ranged from 0–60 minutes for the 15 minutes application and from 0–80 minutes for the 30 minutes application. The magnitude of AL-TENS analgesia ranged between −20% and −36% pain reduction. Furthermore, using an intrasubject design, we observed that a longer application of AL-TENS (30 minutes versus 15 minutes) induced neither longer duration of analgesia nor greater magnitude (greater analgesic effect). This experimental study is the first step leading to the investigation of AL-TENS analgesia duration on a population suffering from clinical pain. The current findings should not be generalized to clinical practice at all. Further studies could also explore precisely the impact of AL-TENS intensity on analgesia. This could help narrow down the best way to use AL-TENS in physical therapy.
